# Repair of the Orbital Wall Fractures in Rabbit Animal Model Using Nanostructured Hydroxyapatite-Based Implant

**DOI:** 10.3390/nano6010011

**Published:** 2016-01-07

**Authors:** Sinziana Gradinaru, Laura Madalina Popescu, Roxana Mioara Piticescu, Sabina Zurac, Radu Ciuluvica, Alexandrina Burlacu, Raluca Tutuianu, Sorina-Nicoleta Valsan, Adrian Mihail Motoc, Liliana Mary Voinea

**Affiliations:** 1 Ophthalmology Department, “Carol Davila” University of Medicine and Pharmacy, 050474 Bucharest, Romania; sanzinici@yahoo.com (S.G.); voineamliliana@yahoo.com (L.M.V.); 2 National R & D Institute for Non-ferrous and Rare Metals, 077145 Ilfov, Romania; mpopescu@imnr.ro (L.M.P.); svalsan@imnr.ro (S.-N.V.); amotoc@imnr.ro (A.M.M.); 3 Pathology Department, “Carol Davila” University of Medicine and Pharmacy, 050474 Bucharest, Romania; 4 Anatomy Department, “Carol Davila” University of Medicine and Pharmacy, 050474 Bucharest, Romania; raduciuluvica@yahoo.com; 5 Institute of Cellular Biology and Pathology “Nicolae Simionescu”, 050568 Bucharest, Romania; sanda.burlacu@icbp.ro (A.B.); raluca.tutuianu@icbp.ro (R.T.)

**Keywords:** nanostructured HAp, mesenchymal stem cells, orbital wall fracture, implant

## Abstract

Cellular uptake and cytotoxicity of nanostructured hydroxyapatite (nanoHAp) are dependent on its physical parameters. Therefore, an understanding of both surface chemistry and morphology of nanoHAp is needed in order to be able to anticipate its *in vivo* behavior. The aim of this paper is to characterize an engineered nanoHAp in terms of physico-chemical properties, biocompatibility, and its capability to reconstitute the orbital wall fractures in rabbits. NanoHAp was synthesized using a high pressure hydrothermal method and characterized by physico-chemical, structural, morphological, and optical techniques. X-ray diffraction revealed HAp crystallites of 21 nm, while Scanning Electron Microscopy (SEM) images showed spherical shapes of HAp powder. Mean particle size of HAp measured by DLS technique was 146.3 nm. Biocompatibility was estimated by the effect of HAp powder on the adhesion and proliferation of mesenchymal stem cells (MSC) in culture. The results showed that cell proliferation on powder-coated slides was between 73.4% and 98.3% of control cells (cells grown in normal culture conditions). Computed tomography analysis of the preformed nanoHAp implanted in orbital wall fractures, performed at one and two months postoperative, demonstrated the integration of the implants in the bones. In conclusion, our engineered nanoHAp is stable, biocompatible, and may be safely considered for reconstruction of orbital wall fractures.

## 1. Introduction

Bone graft materials are widely used for filling bone defects, mostly by orthopedists and oral surgeons; the indications are variable, ranging form fractures to tumors. Lately, several types of biomaterials have been used for grafting, either natural or synthetic, each type of graft presenting specific advantages and disadvantages. In the last ten years, various types of individual implants, such as high-density polyethylene (HDPE), titanium, hydroxyapatite, polydioxanone, and polylactic acid/polyglycolic acid implants have been used for the reconstruction of orbital fractures [1,2,3,4]. This therapeutic reconstruction is very promising due to the excellent anticipated ophthalmologic results [3,5,6,7]. However, there is still no implant able to substitute the functional properties of missing or diseased bone and to sustain and assist cell proliferation and anchorage to the existing bone [8]. Once these requirements are achieved, they will lead to osseointegration and will ensure optimal biomechanical properties of the bone/biomaterial implant [8,9,10].

It is well known that hydroxyapatite (HAp) is one of the most stable bioactive ceramic materials, able to integrate within living tissues and generate excellent biocompatibility. It has been shown that HAp can stimulate the bone growth through osteoconduction mechanism without causing any local or systemic toxicity, inflammation, or body rejection [11,12]. Nontoxicity, lack of inflammatory and immune responses, and high bioactivity makes HAp one of the most used and recommended materials for correction of bone defects.

Recent studies [11,12,13,14,15,16] reported that the morphology of HAp is very important for its successful use in bone regeneration. Thus, HAp was synthesized in different shapes, such as microspheres, plates, nanorods, or nanofibers. Porosity, surface roughness, and mechanical properties are also crucial for HAp use in bone regeneration, as well as the sintering behavior, which can affect several mechanical properties and is directly related to other physical parameters such as particle size and shape [17]. Cellular uptake and cytotoxicity of nanostructured hydroxyapatite (nanoHAp) are dependent on these parameters (size, shape, crystallinity) [18]. It has been shown that nanoHAp might impact cell behavior at both the membrane and the intracellular level. Increased amounts of intracellular calcium may lead to cytotoxic effects as reported in several studies [18]. Therefore, both surface chemistry and morphology are needed to be well understood to anticipate *in vivo* behavior of the bone implant.

NanoHAp powders make the sinterability easier and enhance densification due to an increased surface area:size ratio, which makes them ideal substitutes for natural bone [19]. Up to now, various wet chemical routes in aqueous or non-aqueous solution systems have been employed to synthesize nanostructured HAp [12,20,21], such as hydrothermal [22,23], chemical precipitation [24,25], wet chemical [26,27], and sol–gel methods [28,29].

NanoHAp is currently used as drug delivery material, bone defect filler, or for oral care products in the form of a paste or suspension [18]. One of the potential applications of nanoHAp could be reconstruction of the orbital wall fractures. We report here the synthesis of nanostructured HAp using our previous experience in this field of hydrothermal method in high pressure conditions [30,31,32,33,34], for reconstruction of the orbital wall fractures. To our knowledge, this is the first study reporting the use of a nanostructured HAp for orbital wall fracture reconstruction.

Previous studies in the field of orbital wall regeneration demonstrated the potential of calcium phosphate cement as a useful biomaterial in the reconstruction of the anterior orbital region in a sheep model [4] and ultrahigh molecular weight polyethylene implant as a material for precise reconstruction of orbital wall defects in human patients [3]. A step forward comparing to our previous work [34] is tailoring the experimental parameters in order to obtain a material with controlled structure and properties. The aim of the paper is to characterize engineered nano-hydroxyapatite in terms of its physicochemical properties, *in vitro* behavior, and interactions with living systems for reconstruction of orbital wall fractures.

## 2. Results and Discussion

Obtaining HAp by hydrothermal method has several advantages, such as aqueous reaction medium, nanocrystalline and high purity materials with controlled morphology, and low energy consumption. Physicochemical, structural, and morphological characterizations of nanopowders and sintered pellets were performed in order to have a better understanding of the HAp interaction with living systems, and are presented as follows.

### 2.1. Physicochemical Characterization

Calcium and phosphorus content determined by quantitative analysis, as well as specific surface area and average pore diameter are presented in Table 1.

**Table 1:** Chemical analysis, BET ^1^ surface area and pore size. HAp: Hydroxyapatite.

Sample Type	Ca (%)	P (%)	Ca:P Ratio	BET Specific Surface Area (m^2^/g)	BJH ^2^ Adsorption Average Pore Diameter (nm)
HAp nanopowder	39.34	19.15	1.59	37.11	11.80
HAp sintered at 800 °C/30 min	39.80	19.30	1.60	13.95	8.16

^1^ Brunauer-Emmett-Teller (BET) Surface Area Analysis; ^2^ Barrett-Joyner-Halenda (BJH) Pore Size and Volume Analysis.

### 2.2. Structural Characterization (X-Ray Diffraction)

**Figure 1:**
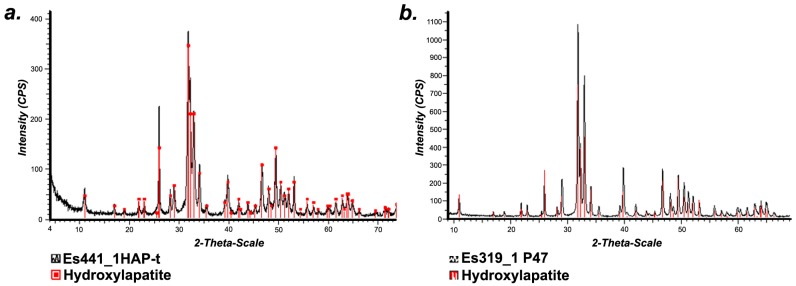
(**a**) X-ray diffractogram of HAp nanopowder; (**b**) X-ray diffractogram of HAp sintered at 800 °C/30 min.

The phase purity and structure were investigated by X-ray diffraction (XRD) analysis. As depicted in Figure 1, XRD analysis revealed a single phase corresponding to Ca_5_(PO_4_)_3_OH with a hexagonal structure, P63/m (176) space group, according to PDF Reference 00-009-0432 (ICDD database, Powder Diffraction File, edited by International Centre for Diffraction Data 2006). Cell parameters were: *a* = 9.42876 Å, *c* = 6.88582 Å, suggesting a preferential growth in the (0 0 l) direction. Crystallite size (Scherrer) was 21.52 nm in the case of HAp powder and 42 nm in the case of sintered pellet.

### 2.3. Morphological Characterization (SEM Analysis)

**Figure 2:**
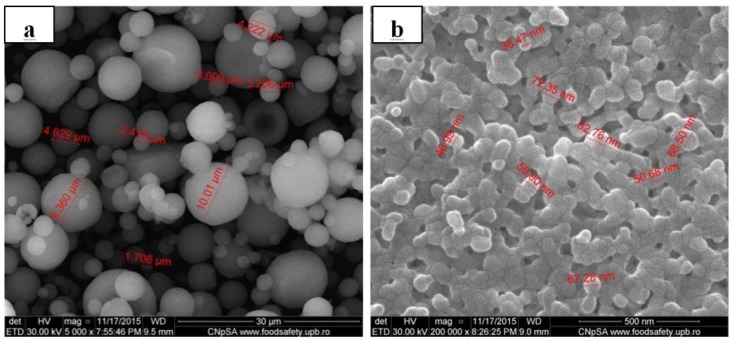
(**a**) Scanning Electron Microscopy (SEM) image of HAp nanopowder; (**b**) SEM image of HAp sintered at 800 °C/30 min.

The SEM image in Figure 2a shows spherical shape of HAp powder prepared by hydrothermal method and granulated in spray dryer. Large agglomerates can be observed with diameters between 2 and 10 µm. After sintering (Figure 2b), compacting and compression deformation of the particles is observed. Diameters of the compressed particles range between 40 and 80 nm.

### 2.4. Particle Size Distribution

**Figure 3:**
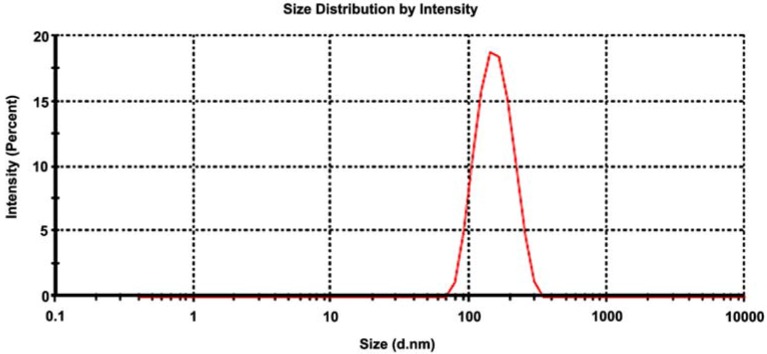
Particle size distribution of HAp nanopowder.

Particle size distribution by intensity of the nanoHAp powder is depicted in Figure 3. Average particle size is 146.3 nm, with a polydispersity index of 6.1%, which suggests a narrow distribution of mean hydrodynamic size.

### 2.5. In Vitro Test

The proliferation of mesenchymal stem cells (MSC) in the presence of HAp was analyzed by incubation of the adhered cells (24 h after seeding) with various dilutions of HAp for four days in culture. The results showed that the two compounds had no cytotoxic effects on cells. Moreover, at higher dilutions (*i.e.*, 1/100 and 1/50), both solutions (HAp1s with pH = 1.33, and HAp2s with pH = 1.88) even stimulated the proliferation of MSC in culture in comparison to normally cultured cells. The increase in MSC proliferation obtained by cell culturing in the presence of low amounts of nanoHAp can only be explained by cell activation. Although the mechanisms leading to such cell activation remains unknown in the absence of other in-depth studies, several other papers have previously reported that stressing environmental conditions (e.g., transient low-pH stressor) might impact the adult stem cells’ behavior by activating several stem cell-specific signaling pathways [35].

**Figure 4:**
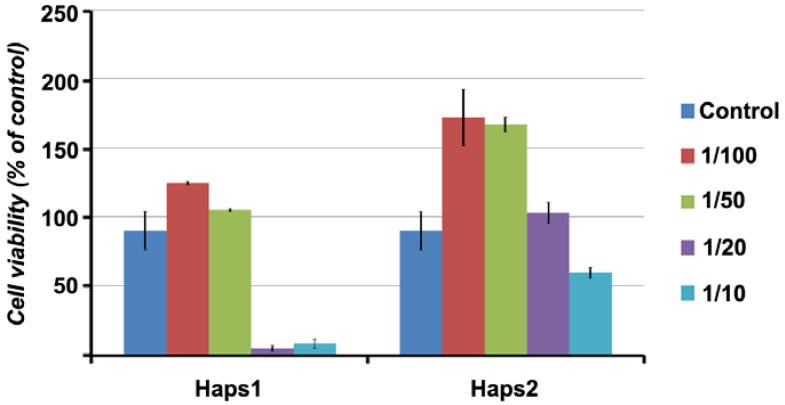
Mesenchymal stem cell (MSC) proliferation in the presence of HAp solubilized in phosphorous acid 5% (pH = 1.31, HAp1s) or phosphorous acid 1% (pH = 1.88, HAp2s). Results are given as mean ± standard deviation (S.D.) of the cell viability expressed as percentage of control cells (cells incubated in normal growth medium in the absence of HAp).

However, the presence of HAp solutions at smaller dilutions in culture medium (*i.e.*, 1/20 and 1/10) resulted in a decrease in cell proliferation (Figure 4). This effect was more pronounced in the presence of HAp2s (pH = 1.31), which is suggested to be a result of acidification of growth medium rather than a direct cytotoxic effect of HAp itself.

When MSC were grown directly on powder HAp-coated slides, cell adhesion and proliferation was between 73.4% and 98.3% (with a mean of 81.8% ± 11.7%, *n* = 6) of cells cultured on uncoated slides, once again confirming the biocompatibility of these materials with *in vitro* cells.

### 2.6. In Vivo Test

Computed tomography (CT) analyses of nanostructured hydroxyapatite implant performed one and two months postoperative revealed time-dependent increasing densities (measured in Hounsfield Units), which are showed in Table 2.

**Table 2:** Maximum density values in Hounsfield Units (HU) of the implant and surrounding tissues regarding normal orbital osseous density.

**Male Rabbit**	**Maximum Density of the Implant One Month Postoperative (HU)**	**Maximum Density of the Implant Two Months Postoperative (HU)**	**Normal Orbital Density (HU)**
Rabbit 1	202	400	900
Rabbit 2	315	450	1000
Rabbit 3	250	402	1120
Rabbit 4	309	409	940
**Male Rabbit**	**Maximum Density of the Surrounding Fibrovascular Tissue One Month Postoperative (HU)**	**Maximum Density of the Surrounding Fibrovascular Tissue Two Months Postoperative (HU)**	**Normal Orbital Density (HU)**
Rabbit 1	40	70	900
Rabbit 2	50	72	1000
Rabbit 3	40	75	1120
Rabbit 4	60	70	940

**Figure 5:**
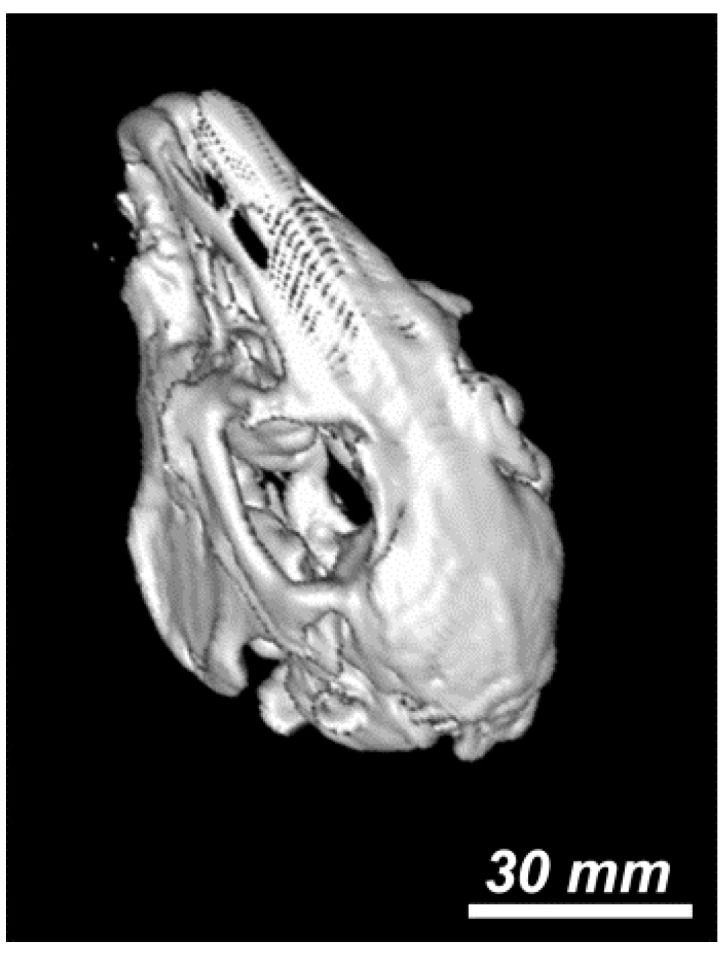
3D computed tomography (CT) reconstruction image of the animal model before surgery. Scale was estimated based on corresponding 2-D image.

**Figure 6:**
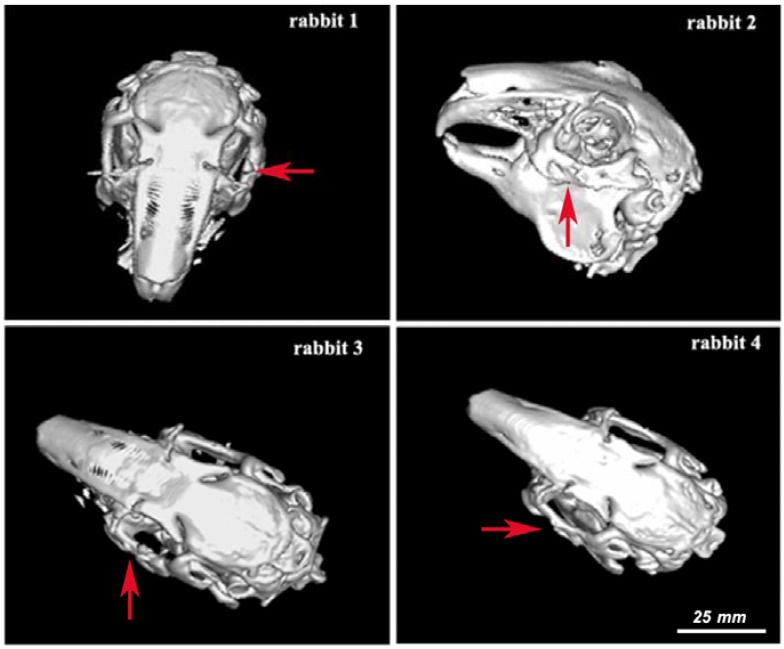
3D CT osseous reconstruction images at one month postoperative for rabbits 1–4. Scale was estimated based on corresponding 2-D image.

**Figure 7:**
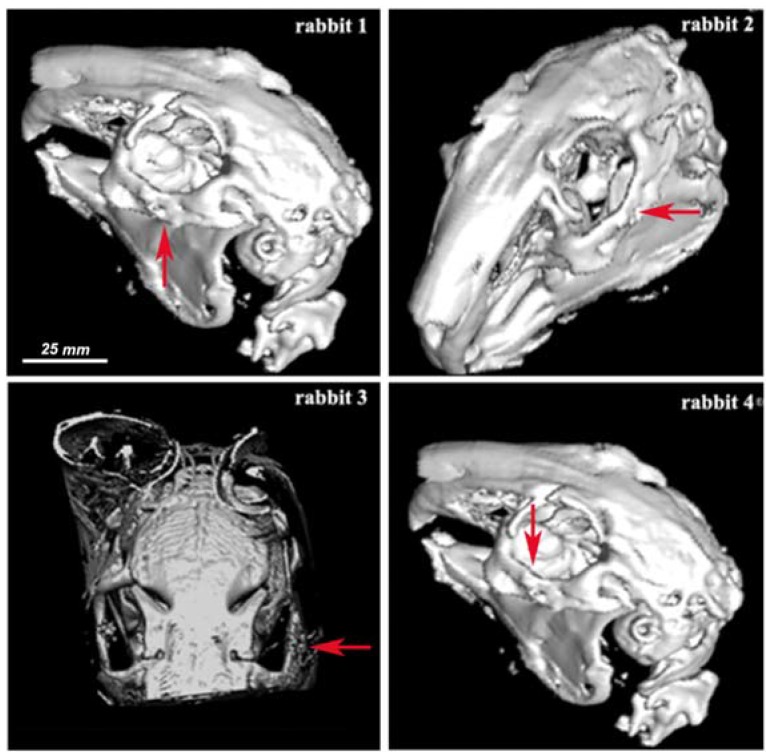
3D CT osseous reconstruction images at two months postoperative for rabbits 1–4. Scale was estimated based on corresponding 2-D image.

The osseous reconstruction images obtained by 3D CT of the animals before and after surgery are presented in Figure 5 (before surgery), Figure 6 (at one month after surgery) and Figure 7 (at two months after surgery). Figure 6 and Figure 7 show the implant of nanoHAp and the surrounding fibrovascular tissue at one and two months postoperative, respectively.

The red arrow marks the implant of nanoHAp. In rabbit 1 (Figure 6), a cleavage plan between the implant and the bone could be noted at one month postoperative, proved by the existence of a surrounding fibrovascular tissue with low density (HU = 40). This cleavage plan was also noted in rabbit 2 between the implant and the bone demonstrated by the presence of a fibrovascular tissue with medium density (HU = 50). For rabbit 3, a large cleavage plan was noted between the implant and the bone. A low density of the surrounding fibrovascular tissue (40 HU) was observed. The explanation might be related to the great difference between the bone density (HU= 1120) and fibrovascular density. The cleavage plan was absent in rabbit 4, as the surrounding fibrovascular tissue had a high density (HU = 60).

At two months postoperative, the cleavage plan was absent in rabbit 1 (Figure 7), but there was still a low-density surrounding fibrovascular tissue. In rabbit 2, there was no cleavage between the implant (red arrow) and the bone. CT scans revealed that the implant had a lower density than the bone at one month post-surgery. However, it progressively increased, so that at two months post-surgery the density of implant appeared similar or even higher than that of the fibrous tissue.

A persistence of a cleavage plan between the implant and the bone, demonstrated by the presence of a low-density fibrovascular tissue (HU = 40), was remarked in two animals at one month postoperative. One of the subjects presented a higher cleavage plan, a possible explanation being related to the greater difference between the density of the normal bone (HU = 1120) and fibrovascular tissue. At two months postoperative the implant had a higher density and the implant was integrated in the osseous tissue.

**Figure 8:**
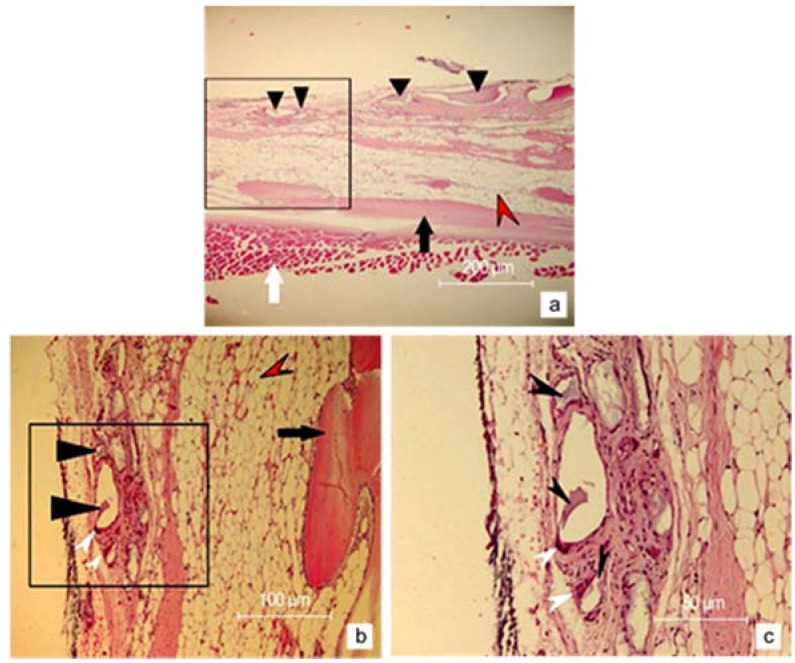
(**a**) Microscopic aspects of HAp implant two months after implantation, as revealed by hematoxilin eosin staining (the magnification is 10×); (**b**) Detailed image of the inset in figure (a) at 20× magnification; (**c**) Detailed image of the inset in figure (b) at 40× magnification. Black arrowheads mark HAp implant. Black arrows mark the osseous tissue. White arrowheads mark osteoclasts. White arrows mark the inferior rectus muscle. Red arrowheads mark the adipose tissue adjacent to bone structure.

The histological analyses showed good integration of the implant in the host tissue, proved by fibrovascular ingrowth between the implant pores. Furthermore, hematoxilin–eosin staining (Figure 8) revealed the presence of the osteoclasts (white arrowheads) with large nuclei at the interface between HAp implant and bone, which demonstrated the degradation of HAp in order to allow the development of new bone.

**Figure 9:**
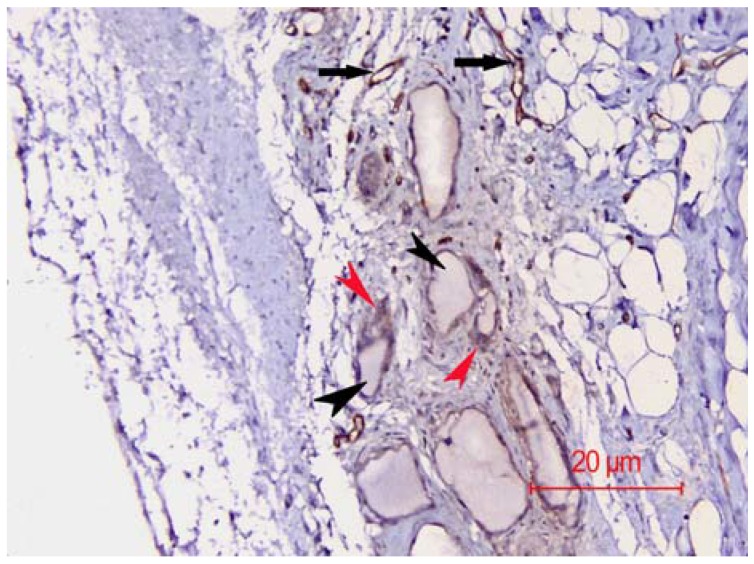
CD31 immunohistochemical staining of the implant. Black arrowheads mark the vascular structures, black arrows mark the CD31-positive cells delineating the vascular structures. Red arrowheads mark the osteoclasts at the interface between the implant and bone. The magnification is 100×.

Immunohistochemical analysis revealed the presence of many vascular structures delineated by CD31^+^-endothelial cells, indicating good vascularization of the implant (Figure 9). In conclusion, our engineered nanoHAp is stable, biocompatible, and may be safely considered for reconstruction of orbital wall fractures.

## 3. Experimental Section

### 3.1. Hydrothermal Synthesis of NanoHAp Powder

Analytical grade precursors, namely calcium nitrate and ammonium dihydrogen phosphate, were solubilized in water under a strict pH control, adding ammonium hydroxide as a mineralizing agent. The aqueous suspension thus obtained was transferred to the Teflon vessel of a closed autoclave (Berghof, Germany) equipped with cooling system, for hydrothermal synthesis at 150 °C/100 barr. The resulted suspension was processed by spray dryer (LabPLANT, Filey, UK). The HAp nanopowder was further evaluated for physical and chemical properties, and the *in vitro* behavior was established by interaction with mouse bone marrow-derived MSC in culture.

### 3.2. Manufacturing of NanoHAp-Based Sintered Pellets

NanoHAp powder, prepared as described above, was mixed with 3% poly(vinyl alcohol) (PVA) as binder and further pressed at 0.5 N/m^2^ using a manual hydraulic press (Specac LTD., Orpington, UK). The resulting pellets, having 6 mm diameter and 1.3 mm height were sintered in air at 800 °C for 30 min, using a CARBOLITE chamber furnace (Hope Valley, UK). Sintering parameters were chosen to provide a specific porosity to the final material (hydroxyapatite implant). These pellets were first evaluated for physical and chemical properties, and then for biocompatibility prior to implantation in rabbits with preformed orbital wall defects for evaluation of their biological effects.

### 3.3. Characterization of NanoHAp

#### 3.3.1. Physico-Chemical Characterization

Chemical composition: Calcium and phosphorus contents in HAp nanopowders were determined by atomic absorption spectroscopy (AAS ZEEnit 700, Analytic Jena AG, Jena, Germany) and inductively-coupled plasma ICP spectrometry (Spectroflame P, Kleve, Germany), respectively.

Specific surface area and porosity measurements: Brunauer–Emmett–Teller (BET) surface area analysis was performed using GEMINI 2380 equipment (Micromeritics Company, Mönchengladbach, Germany) in the following working conditions: nitrogen atmosphere, equilibration time 5 s, saturation pressure 799.570 mmHg.

#### 3.3.2. Structural Characterization (XRD Analysis)

In order to identify the pattern of X-ray diffraction (XRD) for nanoHAp powder, we used a Bruker D8 Advance diffractometer (Karlsruhe, Germany) with monochromatic CuKα radiation (scanning in 2θ range = 4–74°; step size of 0.020 every 2 s).

#### 3.3.3. Morphological Characterization (SEM Analysis)

Morphological observations were carried out by scanning electron microscopy (SEM) using a Quanta Inspect F50 FEG microscope (resolution 1.4 nm) equipped with energy dispersive X-ray detector (EDAX), (FEI Company, Eindhoven, The Netherlands). Particle size measurements have been performed through DigitalMicrograph™, Gatan Inc. (Pleasanton, CA, USA) image acquisition and processing software.

#### 3.3.4. Particle Size Distribution

Particle size distribution was performed by DLS technique, using Zetasizer Nano ZS 90 laser granulometer, Malvern Instruments (Worcestershire, UK), domain 0.6−3.0 µm, temperature range 20–90 °C, dispersion type wet, and specialized software.

### 3.4. Evaluation of Biocompatibility by In Vitro Cell Culture Studies

The *in vitro* behavior of the nanostructured HAp, either as powder layered on the surface of culture cover slides or solubilized in phosphorous acid solution, was assessed by interaction with mouse-derived mesenchymal stem cells (MSC). To this aim, cells were directly seeded onto nanoHAp-coated cover slides and grown for 5 days before viability assessment by 3-(4,5-dimethylthiazol-2-yl)-2,5-diphenyltetrazolium bromide (MTT) assay. The biocompatibility of HAp solubilized in phosphorous acid was evaluated on MSC seeded on 96-well plates (2000 cells/well) in DMEM supplemented with 10% MSC-qualified FCS (fetal calf serum, Invitrogen, Carlsbad, CA, USA). Twenty-four hours later, the medium was replaced with growth medium in the presence of various dilutions of HAp solutions (HAp1s, with a pH of 1.38 and HAp2s with a pH of 1.88) covering the range between 1/5 and 1/100. Cell proliferation was evaluated by MTT assay after 5 days of culture, which represented the period cells usually need to get to confluence.

### 3.5. In Vivo Test

HAp sintered pellets were used as implants in four male rabbits. Before implantation, two bridges were created on the surface of the implants to allow the periostal attachment intraoperatory through a 9-0 vicryl suture.

#### 3.5.1. Animal model

All the procedures were approved by the Ethic Committee of University of Medicine and Pharmacy, code PO-35-F-03/nr.33/08.10.2014, in accordance with the European Convention for the protection of vertebrate animals used for experimental or other scientific purposes (Strasbourg 18.03.1986) and The Directive 2010/63/UE.

#### 3.5.2. Surgical Intervention Methodology

**Figure 10:**
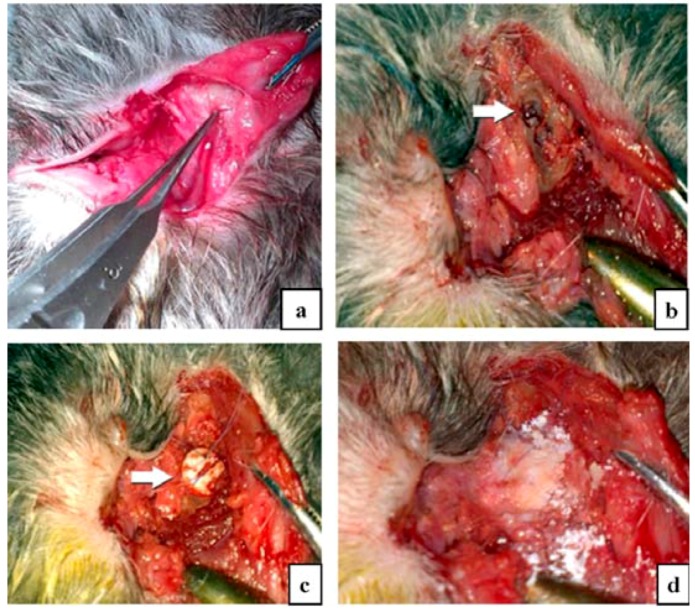
Intra-operatory aspect: (**a**) transconjunctival approach and lateral cantothomy facilitate the inferior rim periosteal elevation; (**b**) the preformed osseous defect in the inferior orbital wall of 0.5 cm diameter (white arrow); (**c**) the orbital implant placed in the defect area and sutured to the periosteum; (**d**) the rest of the defect covered with hydroxyapatite powder.

Four male rabbits (*Oryctolagus cuniculus*) with age around 12 months were weighed then anesthetized by intramuscular administration of Xilazin (Xilazin Bio, Maravet Animal Health) 0.15 mL/kg body weight, and after 10 min by intravenous administration of ketamine (Ketaminol10, MSD Animal Health) 0.1 mL/kg body weight. A volume of 0.15 mL adrenaline 1/100.000 was administered in lateral cantus and inferior conjunctival cul de sac to induce proper local hemostasis. After induction of the general anesthesia, lateral cantothomy and cantholysis were performed and a tractor 4-0 silk thread was passed through the inferior lid to allow visualization of the inferior orbital wall. An inferior conjunctival incision was made in the inferior fornix and the orbicular muscle and orbitary septum were dissected out through inferior orbital margin. After opening the orbitary septum, the inferior rim orbital periosteum was isolated and a 0.5 cm diameter orbital defect was produced in the inferior rim, by using a stomatologic trephine (Figure 10).

The nanostructured implant was placed in the defect and attached to the orbital periosteum with 9-0 vicryl sutures. At the end, the residual defect was filled with nanoHAp powder. The orbital periosteum was reattached and the orbitary septum, the conjunctiva, and the lateral cantus were closed. A 0.3% tobramycin and 0.1% dexamethasone ointment (Tobradex, Alcon, Fort Worth, TX, USA) was applied and subcutaneous enrofloxacine (Ganadexil Enro 5%, Industrial Veterinaria SA, Barcelona, Spain) 0.5 mL/kg bw/day was given for 5 days.

#### 3.5.3. Postoperative Care

Postoperative daily ophthalmological assessment consisting of slit lamp evaluation was conducted for 10 days, and then ophthalmologic evaluations were performed every third day for a month. At one and two months post-surgery, all subjects underwent CT exams under general anesthesia in order to monitor the implant integration. CT scans were performed with a computer tomography Siemens and SOMATOM-SIEMENS software version syngoHEAD CT 2007E. 3D reconstruction was obtained using 3D RECON software. HeadSpi/Fine 0.6-2.4 Sections were used for the orbital examinations.

CT investigation protocol consisted of:

(1) Topogram (selection of the region of interest).
(2) Initial acquisition of 2.4 (avoiding movement artifacts by shortening the exposure and acquisition time).
(3) Reconstruction (HeadSecv 1.2, Headsecv 0.6 and SSD (3D), frontal and sagittal multiplanar reconstruction).
(4) Hounsfield units assessment of the implant and related tissues.

The bone fragment containing the implant was harvested two months after surgery by a similar surgical intervention with a drilling procedure after opening the orbital septum for bone harvesting. The bone was placed in 10% formaldehyde and processed for paraffin embedding and histopathology/immunohistochemistry analysis.

## 4. Conclusions

Crystalline nanoHAp with crystallite size of 21.52 nm was prepared using hydrothermal method in one step, without any further thermal treatment of the obtained powder. *In vitro* test showed that HAp had good biocompatible properties, both as solutions with various dilutions and as powder-coated slides. Moreover, at higher dilutions (*i.e.*, 1/100 and 1/50), HAp solutions even stimulated the proliferation of MSC in culture, in comparison to normally cultured cells. MSC adhesion and proliferation was between 73.4% and 98.3% in the case of powder-coated slides.

The CT analysis of the preformed nanoHAp implant for repairing of the orbital wall fractures showed a good integration of the implant in the bone at two months postoperative, thus demonstrating that our engineered nanoHAp is stable, biocompatible, and may be safely considered for reconstruction of orbital wall fractures.
